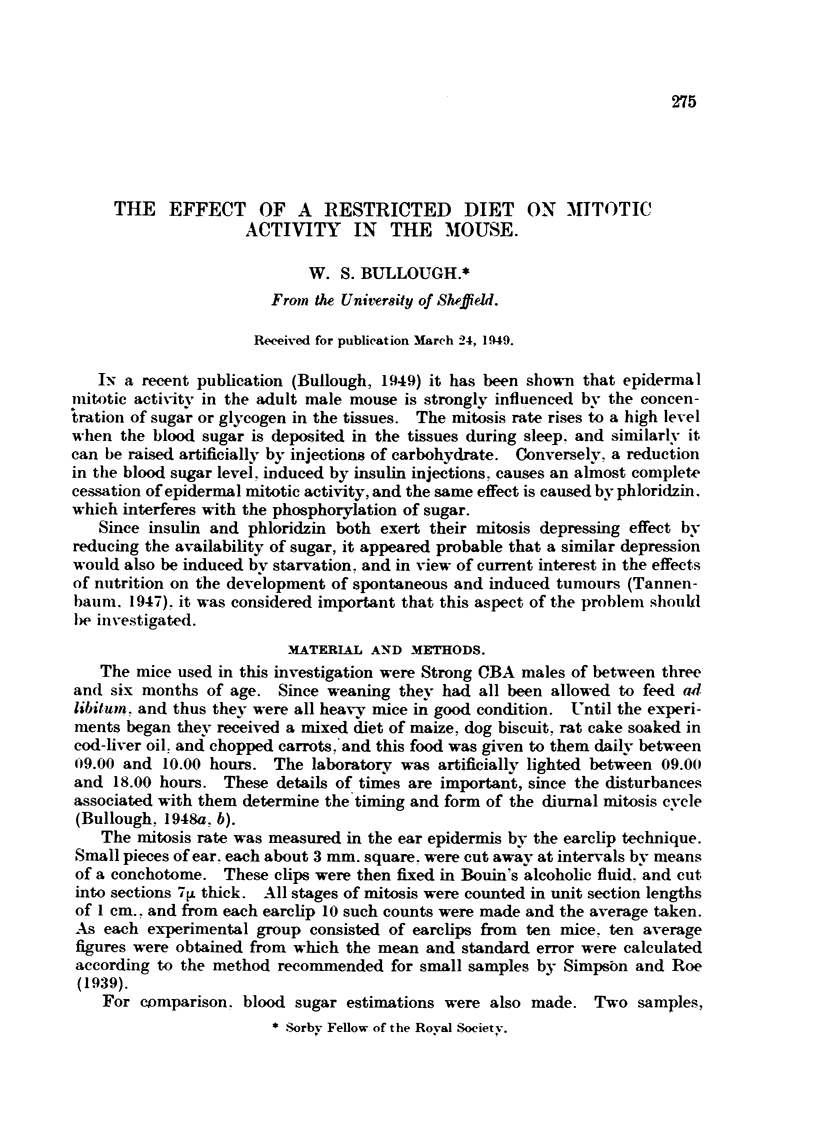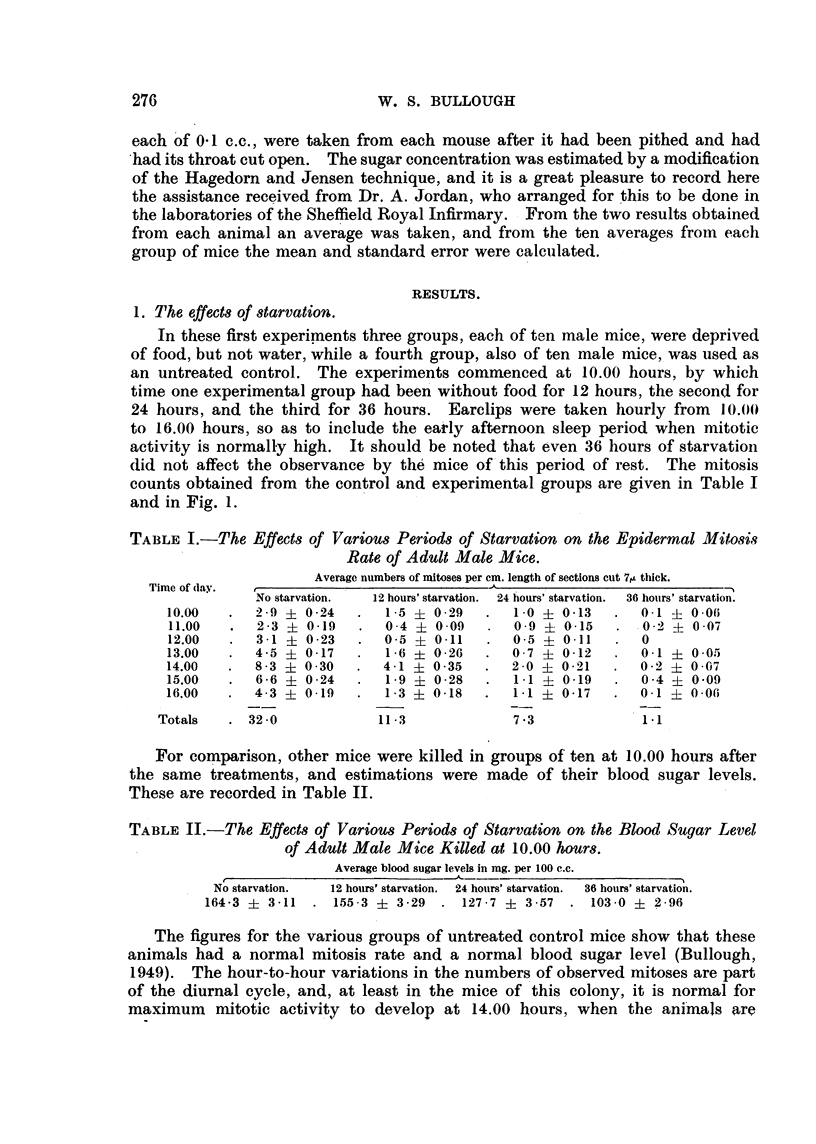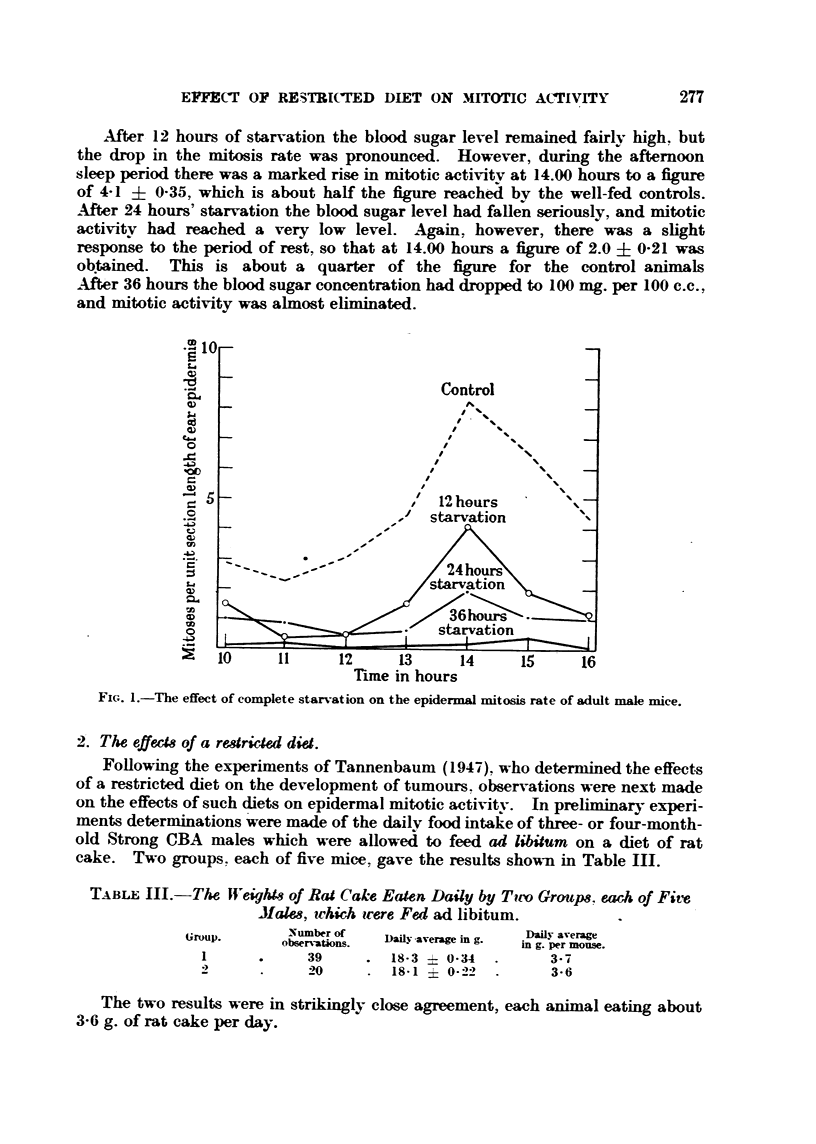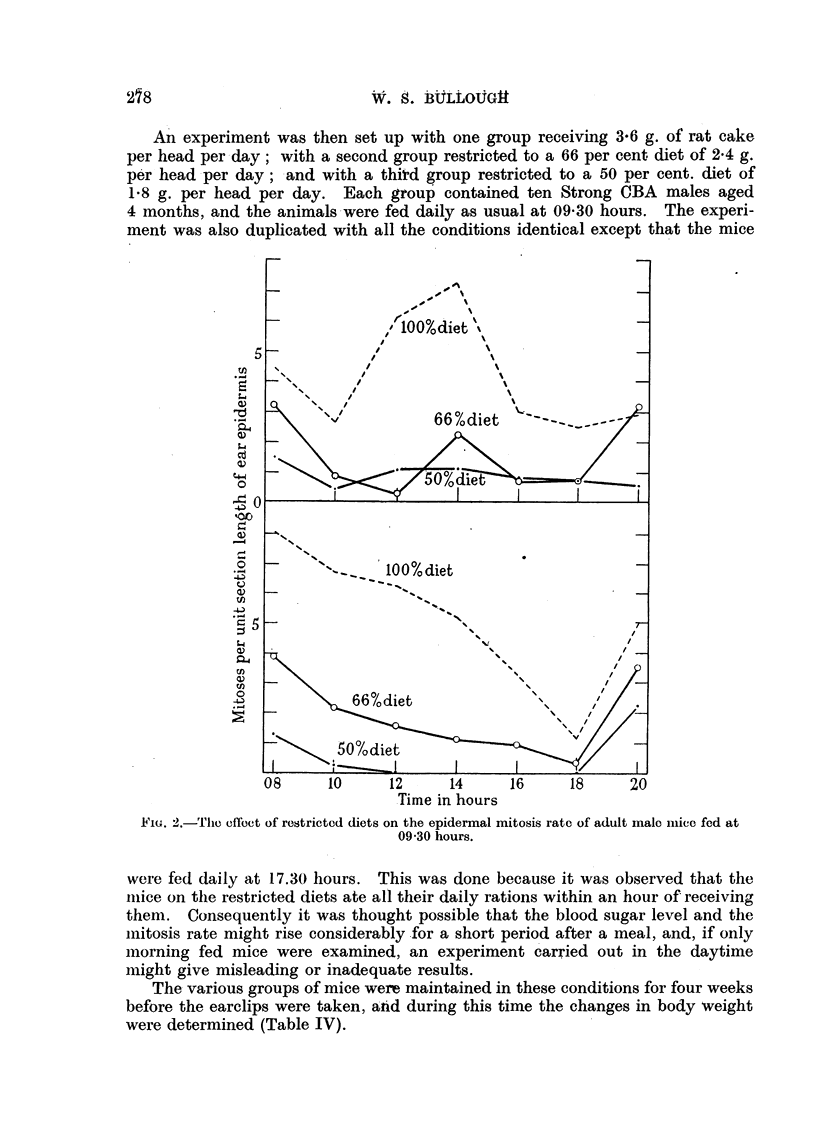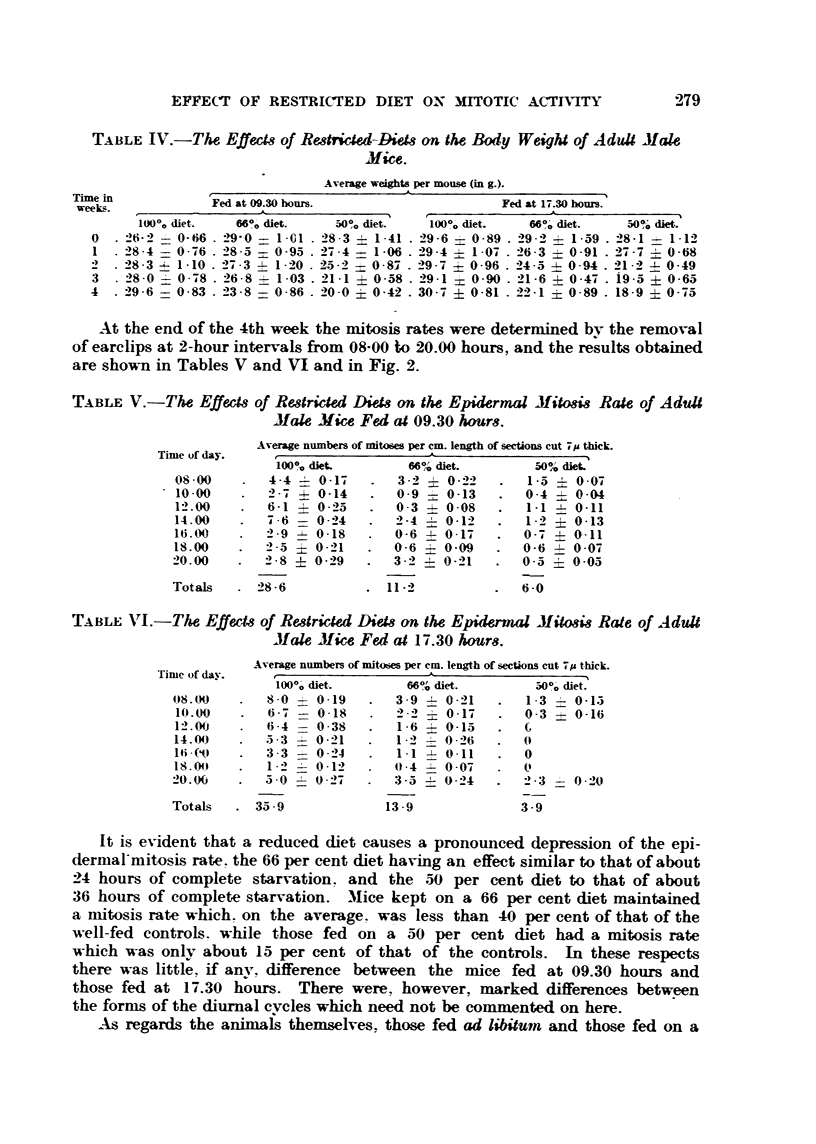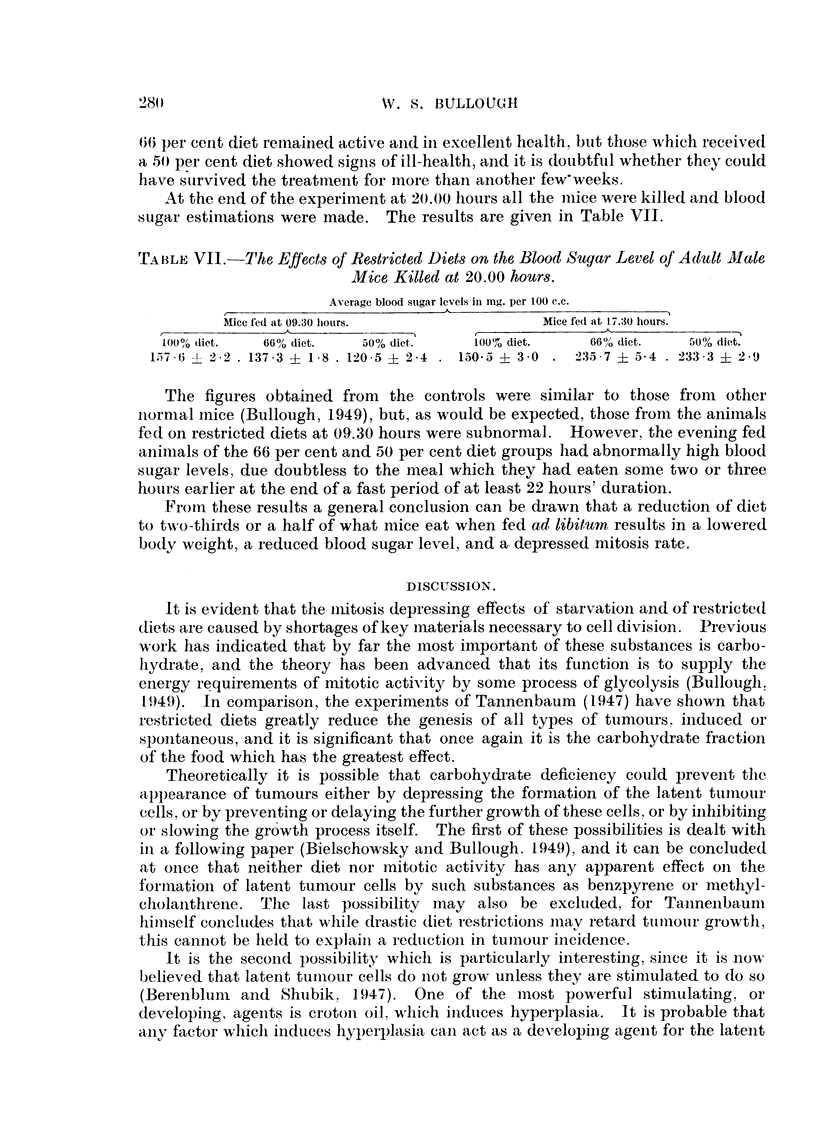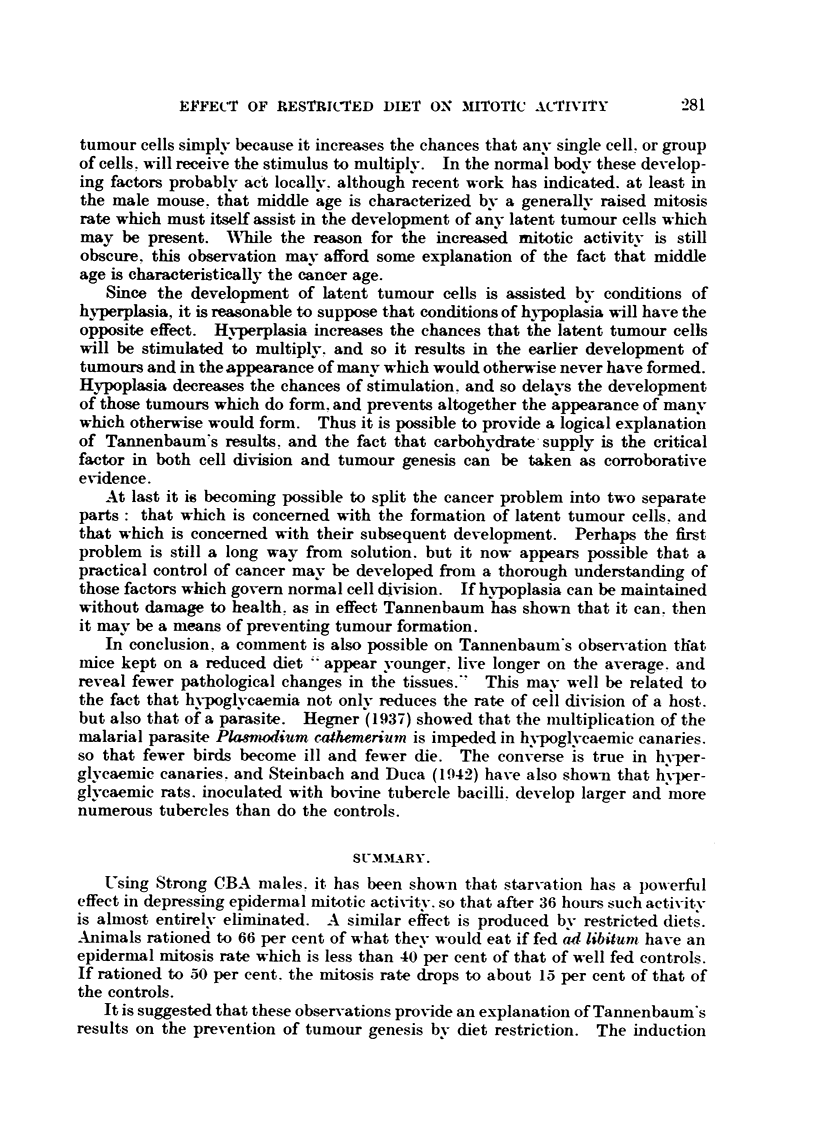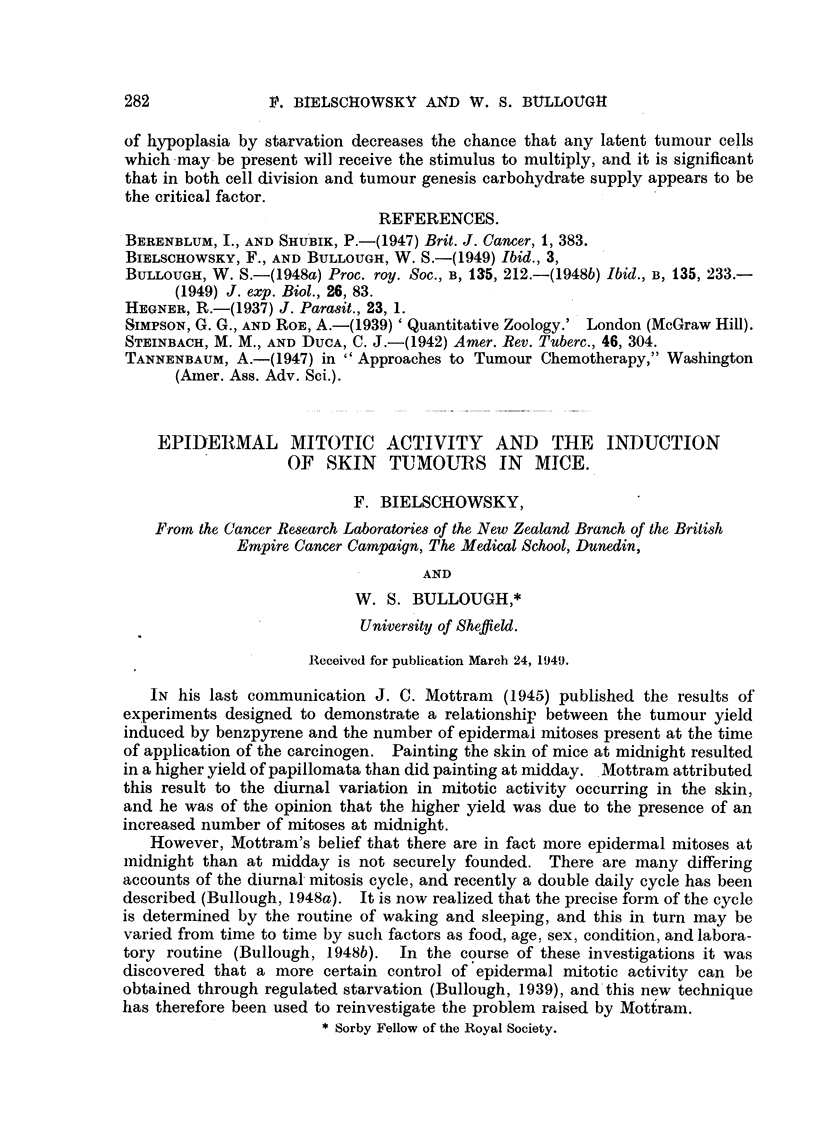# The Effect of a Restricted Diet on Mitotic Activity in the Mouse

**DOI:** 10.1038/bjc.1949.31

**Published:** 1949-06

**Authors:** W. S. Bullough


### THE EFFECT OF A RESTRICTED DIET ON MITOTIC ACTIVITY IN THE MOUSE.

W. S. BULLOUGH.*

*From the University of Sheffield.*

Received for publication March 24, 1949.

In a recent publication (Bullough, 1949) it has been shown that epidermal mitotic activity in the adult male mouse is strongly influenced by the concentration of sugar or glycogen in the tissues. The mitosis rate rises to a high level when the blood sugar is deposited in the tissues during sleep, and similarly it can be raised artificially by injections of carbohydrate. Conversely, a reduction in the blood sugar level, induced by insulin injections, causes an almost complete cessation of epidermal mitotic activity, and the same effect is caused by phloridzin, which interferes with the phosphorylation of sugar.

Since insulin and phloridzin both exert their mitosis depressing effect by reducing the availability of sugar, it appeared probable that a similar depression would also be induced by starvation, and in view of current interest in the effects of nutrition on the development of spontaneous and induced tumours (Tannenbaum, 1947), it was considered important that this aspect of the problem should be investigated.

### MATERIAL AND METHODS.

The mice used in this investigation were Strong CBA males of between three and six months of age. Since weaning they had all been allowed to feed *ad libitum*, and thus they were all heavy mice in good condition. Until the experiments began they received a mixed diet of maize, dog biscuit, rat cake soaked in cod-liver oil, and chopped carrots, and this food was given to them daily between 09.00 and 10.00 hours. The laboratory was artificially lighted between 09.00 and 18.00 hours. These details of times are important, since the disturbances associated with them determine the timing and form of the diurnal mitosis cycle (Bullough, 1948*a*, *b*).

The mitosis rate was measured in the ear epidermis by the earclip technique. Small pieces of ear, each about 3 mm. square, were cut away at intervals by means of a conchotome. These clips were then fixed in Bouin's alcoholic fluid, and cut into sections 7μ thick. All stages of mitosis were counted in unit section lengths of 1 cm., and from each earclip 10 such counts were made and the average taken. As each experimental group consisted of earclips from ten mice, ten average figures were obtained from which the mean and standard error were calculated according to the method recommended for small samples by Simpson and Roe (1939).

For comparison, blood sugar estimations were also made. Two samples,

* Sorby Fellow of the Royal Society.

each of 0·1 c.c., were taken from each mouse after it had been pithed and had had its throat cut open. The sugar concentration was estimated by a modification of the Hagedorn and Jensen technique, and it is a great pleasure to record here the assistance received from Dr. A. Jordan, who arranged for this to be done in the laboratories of the Sheffield Royal Infirmary. From the two results obtained from each animal an average was taken, and from the ten averages from each group of mice the mean and standard error were calculated.

### RESULTS.

### 1. *The effects of starvation.*

In these first experiments three groups, each of ten male mice, were deprived of food, but not water, while a fourth group, also of ten male mice, was used as an untreated control. The experiments commenced at 10.00 hours, by which time one experimental group had been without food for 12 hours, the second for 24 hours, and the third for 36 hours. Earclips were taken hourly from 10.00 to 16.00 hours, so as to include the early afternoon sleep period when mitotic activity is normally high. It should be noted that even 36 hours of starvation did not affect the observance by the mice of this period of rest. The mitosis counts obtained from the control and experimental groups are given in Table I and in Fig. 1.

TABLE I.—*The Effects of Various Periods of Starvation on the Epidermal Mitosis Rate of Adult Male Mice.*

| Time of day. | Average numbers of mitoses per cm. length of sections cut 7μ thick. | | | |
|---|---|---|---|---|
| | No starvation. | 12 hours' starvation. | 24 hours' starvation. | 36 hours' starvation. |
| 10.00 | 2·9 ± 0·24 | 1·5 ± 0·29 | 1·0 ± 0·13 | 0·1 ± 0·06 |
| 11.00 | 2·3 ± 0·19 | 0·4 ± 0·09 | 0·9 ± 0·15 | 0·2 ± 0·07 |
| 12.00 | 3·1 ± 0·23 | 0·5 ± 0·11 | 0·5 ± 0·11 | 0 |
| 13.00 | 4·5 ± 0·17 | 1·6 ± 0·26 | 0·7 ± 0·12 | 0·1 ± 0·05 |
| 14.00 | 8·3 ± 0·30 | 4·1 ± 0·35 | 2·0 ± 0·21 | 0·2 ± 0·07 |
| 15.00 | 6·6 ± 0·24 | 1·9 ± 0·28 | 1·1 ± 0·19 | 0·4 ± 0·09 |
| 16.00 | 4·3 ± 0·19 | 1·3 ± 0·18 | 1·1 ± 0·17 | 0·1 ± 0·06 |
| Totals | 32·0 | 11·3 | 7·3 | 1·1 |

For comparison, other mice were killed in groups of ten at 10.00 hours after the same treatments, and estimations were made of their blood sugar levels. These are recorded in Table II.

TABLE II.—*The Effects of Various Periods of Starvation on the Blood Sugar Level of Adult Male Mice Killed at* 10.00 *hours.*

| Average blood sugar levels in mg. per 100 c.c. | | | |
|---|---|---|---|
| No starvation. | 12 hours' starvation. | 24 hours' starvation. | 36 hours' starvation. |
| 164·3 ± 3·11 | 155·3 ± 3·29 | 127·7 ± 3·57 | 103·0 ± 2·96 |

The figures for the various groups of untreated control mice show that these animals had a normal mitosis rate and a normal blood sugar level (Bullough, 1949). The hour-to-hour variations in the numbers of observed mitoses are part of the diurnal cycle, and, at least in the mice of this colony, it is normal for maximum mitotic activity to develop at 14.00 hours, when the animals are

After 12 hours of starvation the blood sugar level remained fairly high, but the drop in the mitosis rate was pronounced. However, during the afternoon sleep period there was a marked rise in mitotic activity at 14.00 hours to a figure of 4·1 ± 0·35, which is about half the figure reached by the well-fed controls. After 24 hours' starvation the blood sugar level had fallen seriously, and mitotic activity had reached a very low level. Again, however, there was a slight response to the period of rest, so that at 14.00 hours a figure of 2.0 ± 0·21 was obtained. This is about a quarter of the figure for the control animals After 36 hours the blood sugar concentration had dropped to 100 mg. per 100 c.c., and mitotic activity was almost eliminated.

FIG. 1.—The effect of complete starvation on the epidermal mitosis rate of adult male mice.

2. *The effects of a restricted diet.*

Following the experiments of Tannenbaum (1947), who determined the effects of a restricted diet on the development of tumours, observations were next made on the effects of such diets on epidermal mitotic activity. In preliminary experiments determinations were made of the daily food intake of three- or four-month-old Strong CBA males which were allowed to feed *ad libitum* on a diet of rat cake. Two groups, each of five mice, gave the results shown in Table III.

TABLE III.—*The Weights of Rat Cake Eaten Daily by Two Groups, each of Five Males, which were Fed* ad libitum.

| Group. | Number of observations. | Daily average in g. | Daily average in g. per mouse. |
|---|---|---|---|
| 1 | 39 | 18·3 ± 0·34 | 3·7 |
| 2 | 20 | 18·1 ± 0·22 | 3·6 |

The two results were in strikingly close agreement, each animal eating about 3·6 g. of rat cake per day.

An experiment was then set up with one group receiving 3·6 g. of rat cake per head per day ; with a second group restricted to a 66 per cent diet of 2·4 g. per head per day ; and with a third group restricted to a 50 per cent. diet of 1·8 g. per head per day. Each group contained ten Strong CBA males aged 4 months, and the animals were fed daily as usual at 09·30 hours. The experiment was also duplicated with all the conditions identical except that the mice

FIG. 2.—The effect of restricted diets on the epidermal mitosis rate of adult male mice fed at 09·30 hours.

were fed daily at 17.30 hours. This was done because it was observed that the mice on the restricted diets ate all their daily rations within an hour of receiving them. Consequently it was thought possible that the blood sugar level and the mitosis rate might rise considerably for a short period after a meal, and, if only morning fed mice were examined, an experiment carried out in the daytime might give misleading or inadequate results.

The various groups of mice were maintained in these conditions for four weeks before the earclips were taken, and during this time the changes in body weight were determined (Table IV).

TABLE IV.—*The Effects of Restricted Diets on the Body Weight of Adult Male Mice.*

| Time in weeks. | Average weights per mouse (in g.). Fed at 09.30 hours. | | | Average weights per mouse (in g.). Fed at 17.30 hours. | | |
|---|---|---|---|---|---|---|
| | 100% diet. | 66% diet. | 50% diet. | 100% diet. | 66% diet. | 50% diet. |
| 0 | 26·2 ± 0·66 | 29·0 ± 1·01 | 28·3 ± 1·41 | 29·6 ± 0·89 | 29·2 ± 1·59 | 28·1 ± 1·12 |
| 1 | 28·4 ± 0·76 | 28·5 ± 0·95 | 27·4 ± 1·06 | 29·4 ± 1·07 | 26·3 ± 0·91 | 27·7 ± 0·68 |
| 2 | 28·3 ± 1·10 | 27·3 ± 1·20 | 25·2 ± 0·87 | 29·7 ± 0·96 | 24·5 ± 0·94 | 21·2 ± 0·49 |
| 3 | 28·0 ± 0·78 | 26·8 ± 1·03 | 21·1 ± 0·58 | 29·1 ± 0·90 | 21·6 ± 0·47 | 19·5 ± 0·65 |
| 4 | 29·6 ± 0·83 | 23·8 ± 0·86 | 20·0 ± 0·42 | 30·7 ± 0·81 | 22·1 ± 0·89 | 18·9 ± 0·75 |

At the end of the 4th week the mitosis rates were determined by the removal of earclips at 2-hour intervals from 08·00 to 20.00 hours, and the results obtained are shown in Tables V and VI and in Fig. 2.

TABLE V.—*The Effects of Restricted Diets on the Epidermal Mitosis Rate of Adult Male Mice Fed at* 09.30 *hours.*

| Time of day. | Average numbers of mitoses per cm. length of sections cut 7 μ thick. 100% diet. | 66% diet. | 50% diet. |
|---|---|---|---|
| 08·00 | 4·4 ± 0·17 | 3·2 ± 0·22 | 1·5 ± 0·07 |
| 10·00 | 2·7 ± 0·14 | 0·9 ± 0·13 | 0·4 ± 0·04 |
| 12.00 | 6·1 ± 0·25 | 0·3 ± 0·08 | 1·1 ± 0·11 |
| 14.00 | 7·6 ± 0·24 | 2·4 ± 0·12 | 1·2 ± 0·13 |
| 16.00 | 2·9 ± 0·18 | 0·6 ± 0·17 | 0·7 ± 0·11 |
| 18.00 | 2·5 ± 0·21 | 0·6 ± 0·09 | 0·6 ± 0·07 |
| 20.00 | 2·8 ± 0·29 | 3·2 ± 0·21 | 0·5 ± 0·05 |
| Totals | 28·6 | 11·2 | 6·0 |

TABLE VI.—*The Effects of Restricted Diets on the Epidermal Mitosis Rate of Adult Male Mice Fed at* 17.30 *hours.*

| Time of day. | Average numbers of mitoses per cm. length of sections cut 7 μ thick. 100% diet. | 66% diet. | 50% diet. |
|---|---|---|---|
| 08.00 | 8·0 ± 0·19 | 3·9 ± 0·21 | 1·3 ± 0·15 |
| 10.00 | 6·7 ± 0·18 | 2·2 ± 0·17 | 0·3 ± 0·16 |
| 12.00 | 6·4 ± 0·38 | 1·6 ± 0·15 | 0 |
| 14.00 | 5·3 ± 0·21 | 1·2 ± 0·26 | 0 |
| 16·00 | 3·3 ± 0·24 | 1·1 ± 0·11 | 0 |
| 18.00 | 1·2 ± 0·12 | 0·4 ± 0·07 | 0 |
| 20.00 | 5·0 ± 0·27 | 3·5 ± 0·24 | 2·3 ± 0·20 |
| Totals | 35·9 | 13·9 | 3·9 |

It is evident that a reduced diet causes a pronounced depression of the epidermal mitosis rate, the 66 per cent diet having an effect similar to that of about 24 hours of complete starvation, and the 50 per cent diet to that of about 36 hours of complete starvation. Mice kept on a 66 per cent diet maintained a mitosis rate which, on the average, was less than 40 per cent of that of the well-fed controls, while those fed on a 50 per cent diet had a mitosis rate which was only about 15 per cent of that of the controls. In these respects there was little, if any, difference between the mice fed at 09.30 hours and those fed at 17.30 hours. There were, however, marked differences between the forms of the diurnal cycles which need not be commented on here.

As regards the animals themselves, those fed *ad libitum* and those fed on a

66 per cent diet remained active and in excellent health, but those which received a 50 per cent diet showed signs of ill-health, and it is doubtful whether they could have survived the treatment for more than another few weeks.

At the end of the experiment at 20.00 hours all the mice were killed and blood sugar estimations were made. The results are given in Table VII.

TABLE VII.—*The Effects of Restricted Diets on the Blood Sugar Level of Adult Male Mice Killed at* 20.00 *hours.*

| Average blood sugar levels in mg. per 100 c.c. | | | | | |
|---|---|---|---|---|---|
| Mice fed at 09.30 hours. | | | Mice fed at 17.30 hours. | | |
| 100% diet. | 66% diet. | 50% diet. | 100% diet. | 66% diet. | 50% diet. |
| 157·6 ± 2·2 | 137·3 ± 1·8 | 120·5 ± 2·4 | 150·5 ± 3·0 | 235·7 ± 5·4 | 233·3 ± 2·9 |

The figures obtained from the controls were similar to those from other normal mice (Bullough, 1949), but, as would be expected, those from the animals fed on restricted diets at 09.30 hours were subnormal. However, the evening fed animals of the 66 per cent and 50 per cent diet groups had abnormally high blood sugar levels, due doubtless to the meal which they had eaten some two or three hours earlier at the end of a fast period of at least 22 hours' duration.

From these results a general conclusion can be drawn that a reduction of diet to two-thirds or a half of what mice eat when fed *ad libitum* results in a lowered body weight, a reduced blood sugar level, and a depressed mitosis rate.

### DISCUSSION.

It is evident that the mitosis depressing effects of starvation and of restricted diets are caused by shortages of key materials necessary to cell division. Previous work has indicated that by far the most important of these substances is carbohydrate, and the theory has been advanced that its function is to supply the energy requirements of mitotic activity by some process of glycolysis (Bullough, 1949). In comparison, the experiments of Tannenbaum (1947) have shown that restricted diets greatly reduce the genesis of all types of tumours, induced or spontaneous, and it is significant that once again it is the carbohydrate fraction of the food which has the greatest effect.

Theoretically it is possible that carbohydrate deficiency could prevent the appearance of tumours either by depressing the formation of the latent tumour cells, or by preventing or delaying the further growth of these cells, or by inhibiting or slowing the growth process itself. The first of these possibilities is dealt with in a following paper (Bielschowsky and Bullough, 1949), and it can be concluded at once that neither diet nor mitotic activity has any apparent effect on the formation of latent tumour cells by such substances as benzpyrene or methylcholanthrene. The last possibility may also be excluded, for Tannenbaum himself concludes that while drastic diet restrictions may retard tumour growth, this cannot be held to explain a reduction in tumour incidence.

It is the second possibility which is particularly interesting, since it is now believed that latent tumour cells do not grow unless they are stimulated to do so (Berenblum and Shubik, 1947). One of the most powerful stimulating, or developing, agents is croton oil, which induces hyperplasia. It is probable that any factor which induces hyperplasia can act as a developing agent for the latent tumour cells simply because it increases the chances that any single cell, or group of cells, will receive the stimulus to multiply. In the normal body these developing factors probably act locally, although recent work has indicated, at least in the male mouse, that middle age is characterized by a generally raised mitosis rate which must itself assist in the development of any latent tumour cells which may be present. While the reason for the increased mitotic activity is still obscure, this observation may afford some explanation of the fact that middle age is characteristically the cancer age.

Since the development of latent tumour cells is assisted by conditions of hyperplasia, it is reasonable to suppose that conditions of hypoplasia will have the opposite effect. Hyperplasia increases the chances that the latent tumour cells will be stimulated to multiply, and so it results in the earlier development of tumours and in the appearance of many which would otherwise never have formed. Hypoplasia decreases the chances of stimulation, and so delays the development of those tumours which do form, and prevents altogether the appearance of many which otherwise would form. Thus it is possible to provide a logical explanation of Tannenbaum's results, and the fact that carbohydrate supply is the critical factor in both cell division and tumour genesis can be taken as corroborative evidence.

At last it is becoming possible to split the cancer problem into two separate parts : that which is concerned with the formation of latent tumour cells, and that which is concerned with their subsequent development. Perhaps the first problem is still a long way from solution, but it now appears possible that a practical control of cancer may be developed from a thorough understanding of those factors which govern normal cell division. If hypoplasia can be maintained without damage to health, as in effect Tannenbaum has shown that it can, then it may be a means of preventing tumour formation.

In conclusion, a comment is also possible on Tannenbaum's observation that mice kept on a reduced diet "appear younger, live longer on the average, and reveal fewer pathological changes in the tissues." This may well be related to the fact that hypoglycaemia not only reduces the rate of cell division of a host, but also that of a parasite. Hegner (1937) showed that the multiplication of the malarial parasite *Plasmodium cathemerium* is impeded in hypoglycaemic canaries, so that fewer birds become ill and fewer die. The converse is true in hyperglycaemic canaries, and Steinbach and Duca (1942) have also shown that hyperglycaemic rats, inoculated with bovine tubercle bacilli, develop larger and more numerous tubercles than do the controls.

### SUMMARY.

Using Strong CBA males, it has been shown that starvation has a powerful effect in depressing epidermal mitotic activity, so that after 36 hours such activity is almost entirely eliminated. A similar effect is produced by restricted diets. Animals rationed to 66 per cent of what they would eat if fed *ad libitum* have an epidermal mitosis rate which is less than 40 per cent of that of well fed controls. If rationed to 50 per cent, the mitosis rate drops to about 15 per cent of that of the controls.

It is suggested that these observations provide an explanation of Tannenbaum's results on the prevention of tumour genesis by diet restriction. The induction of hypoplasia by starvation decreases the chance that any latent tumour cells which may be present will receive the stimulus to multiply, and it is significant that in both cell division and tumour genesis carbohydrate supply appears to be the critical factor.

REFERENCES.

BERENBLUM, I., AND SHUBIK, P.—(1947) *Brit. J. Cancer*, **1**, 383.
BIELSCHOWSKY, F., AND BULLOUGH, W. S.—(1949) *Ibid.*, **3**,
BULLOUGH, W. S.—(1948*a*) *Proc. roy. Soc.*, B, **135**, 212.—(1948*b*) *Ibid.*, B, **135**, 233.—(1949) *J. exp. Biol.*, **26**, 83.
HEGNER, R.—(1937) *J. Parasit.*, **23**, 1.
SIMPSON, G. G., AND ROE, A.—(1939) ' Quantitative Zoology.' London (McGraw Hill).
STEINBACH, M. M., AND DUCA, C. J.—(1942) *Amer. Rev. Tuberc.*, **46**, 304.
TANNENBAUM, A.—(1947) in " Approaches to Tumour Chemotherapy," Washington (Amer. Ass. Adv. Sci.).